# Sclareol and cinnarizine are non-selective inhibitors of voltage-gated Cav1.3 L-type Ca^2+^ channels

**DOI:** 10.1080/19336950.2025.2556101

**Published:** 2025-09-16

**Authors:** Lucia Zanetti, Ferenc Török, Luisa Leitzbach, Holger Stark, Jörg Striessnig

**Affiliations:** a Department of Pharmacology and Toxicology, Institute of Pharmacy, Center for Molecular Biosciences Innsbruck, University of Innsbruck, Innsbruck, Austria; b Institute of Pharmaceutical and Medicinal Chemistry, Heinrich Heine University Düsseldorf, Duesseldorf, Germany

**Keywords:** L-type calcium channels, sclareol, Parkinson’s disease, calcium channel blockers, cinnarizine

## Abstract

A growing body of preclinical evidence indicates that the inhibition of voltage-gated Cav1.3 L-type Ca^2+^ channels could be a therapeutic concept for the therapy of treatment-resistant hypertension, spinal injury and for neuroprotection in early Parkinson’s disease (PD). However, available Ca^2+^-channel blockers are potent inhibitors of vascular Cav1.2 L-type channels which can cause low blood pressure as an adverse drug reaction. Therefore, Cav1.3-selective inhibitors are needed to further investigate the therapeutic potential of Cav1.3 as drug target in vivo. The bicyclic diterpene alcohol sclareol has recently been reported to exert neuroprotective properties in a mouse PD model by blocking Cav1.3 L-type channels. This study investigates the proposed Cav1.3-selectivity of sclareol compared to Cav1.2 and to other voltage-gated Ca^2+^ channels in whole-cell patch-clamp experiments. Various stimulation protocols, including dopamine neuron-like firing patterns show that sclareol is neither a subtype-selective nor a potent blocker of heterologously expressed Cav1.3 and inhibits also Cav2.3 channels. Therefore, the contribution of Cav1.3 channel inhibition for the previously reported neuroprotective effects of sclareol in a mouse PD model remains unclear. In addition, cinnarizine, a vertigo therapeutic also under investigation for inhibition of Cav1.3-mediated aldosterone-secretion, inhibits Cav1.3 channels in a frequency-dependent manner, but also without relevant selectivity with respect to Cav1.3.

## Introduction

Voltage-gated calcium channels (VGCCs) play a crucial role in a variety of physiological processes, including hormone secretion, muscle contraction, gene expression, neurotransmitter release and different types of learning and memory [1]. The selective targeting of Cav1.3 channels, a member of the dihydropyridine (DHP)-sensitive L-type channel family (Cav1 [2]), has attracted increasing attention in recent years due to its involvement in pathophysiological processes of potential relevance for human disease. Cav1.3 signaling contributes to the high vulnerability of substantia nigra (SN) dopamine neurons to degeneration in Parkinson’s disease (PD) [3–5]. In addition, inhibition of Cav1.3-channels reduces spasticity in a preclinical model of spinal cord injury [6], may improve symptoms in a neurodevelopmental syndrome caused by Cav1.3 (*CACNA1D*) gain of function mutations [7] and is under investigation for reducing aldosterone secretion in primary hyperaldosteronism [8]. The clinically used Ca^2+^ channel blockers, such as DHPs, verapamil and diltiazem, exert their cardiovascular therapeutic effects primarily by inhibiting Cav1.2 channels in arterial smooth muscle and in the heart [1]. Since these drugs are not Cav1.3-selective, side effects from blood pressure lowering and from cardiodepression through Cav1.2 limits their use for the efficient inhibition of Cav1.3 [9]. So far, the development of pharmacological agents selectively targeting Cav1.3 channels has been hampered by the high degree of sequence homology between the pore-forming Cav1.3 and Cav1.2 α1-subunits [10]. As a result, there remains a significant need for the development of selective Cav1.3 modulators as specific tools to probe their pharmacotherapeutic potential in non-clinical studies.

Recently, the natural compound sclareol has been identified as a Cav1.3 inhibitor in an elegant cell-based assay in which reporter gene expression is driven by Ca^2+^ channel activity [11]. Moreover, in a mouse model of PD, sclareol treatment protected SN dopamine neurons from degeneration [11]. However, so far sclareol selectivity has not been rigorously tested in whole-cell patch-clamp studies, which allows the direct quantification of drug potency on Ca^2+^ current under defined electrical activity patterns. This enables a straightforward analysis of potential voltage- or frequency-dependent effects and of its potency during SN dopamine neuron-like waveforms. We therefore conducted whole-cell voltage-clamp experiments to quantify sclareol potency for Cav1.3 and its selectivity toward Cav1.2 and Cav2.3, another channel subtype potentially involved in PD pathophysiology [12]. We also included the L-type channel blocker cinnarizine [13] into our analysis. Cinnarizine, a drug approved for the treatment of vertigo, has been shown to bind within the Cav1.3 channel pore in a cryo-EM study [10]. It was therefore also explored as a promising Cav1.3 inhibitor in a clinical trial in patients with primary aldosteronism [8], but its proposed selectivity for Cav1.3 versus Cav1.2 has never been investigated. We show that sclareol is neither a potent nor a selective inhibitor of Cav1.3 and its potency is not measurably affected by voltage or stimulation frequency. Cinnarizine inhibits Cav1.3 and Cav1.2 channels also with similar potency but in a frequency-dependent manner.

## Materials and methods

### Chemical compounds

Chemical compounds studied in this article: Sclareol (PubChem CID: 163263); Cinnarizine (PubChem CID: 1547484); Isradipine (PubChem CID: 3784).

### Cell culture and transfection

Pharmacological activity of sclareol on human Cav1.2 [14], Cav1.3_S_ or human Cav1.3_L_ [15] (accession number: EU_363339) was tested in tsA-201 cells stably expressing the respective α1 subunit together with auxiliary β3 (rat, NM_012828) and α2δ1 (rabbit, NM_001082276) subunits [14] upon induction with 1 µg/mL doxycycline (catalog #D1822, Sigma-Aldrich). Cells were used for experiments 20–72 h after induction. For experiments with transiently expressed channels, including Cav2.3 and all cinnarizine drug recordings, tsA-201 cells were transfected with 3 μg Cav2.3e α1 (cloned into pcDNA3 [12,16]), Cav1.2 α1 or Cav1.3_L_ α1 together with 2 μg β3 and 2.5 μg α2δ1-subunits using the Ca^2+^-phosphate precipitation technique together with 1.5 μg eGFP as a transfection marker. TsA-201 cells (HEK-293 cell line stably expressing SV40 temperature-sensitive T-antigen) obtained from the European Collection of Authenticated Cell Cultures (ECACC, catalog number 96,121,229, lot number 13D034) were cultured in Dulbecco’s modified Eagle’s medium (DMEM; Sigma-Aldrich, D6546) completed with 10% FBS (Gibco 10,270–106), 2 mM L-glutamine (Gibco 25,030–032), penicillin (10 U/ml; Sigma-Aldrich, P3032) and streptomycin (10 µg/ml; Sigma-Aldrich, S6501) at 37°C and 5% CO_2_ in a humidified incubator between passage number 8 and 20 for transfected cells, and number 20 to 100 for stable cell lines. All data were obtained from at least 3 independent transfections or induction rounds.

### Electrophysiological recordings

Whole-cell patch-clamp experiments were performed independently on different setups by two researchers at room temperature (20–24°C) using Axopatch 200B amplifiers (Axon Instruments) and digitizers (Digi-data, 1322A digitizer, Molecular Devices, San José, CA, USA). Recordings were sampled at 20 or 50 kHz, low-pass filtered at 1–2 kHz and compensated for 60–99% of the series resistance. Recording solutions contained (in mM): bath solution for stable transfection: 2 CaCl_2_, 10 HEPES, 170 Choline-Cl, 1 MgCl_2_, adjusted to pH 7.3 with CsOH; bath solution for transient transfection: 15 CaCl_2_, 10 HEPES, 150 Choline-Cl, 1 MgCl_2_, adjusted to pH 7.3 with CsOH; pipette solution: 135 CsCl, 10 Cs-EGTA, 1 MgCl_2_, 10 HEPES, 4 Na_2_ATP, adjusted to pH 7.3 with CsOH. The voltage dependence of activation (I-V curve) was obtained by applying 25 ms-long square pulse to increasing test potentials (5 mV steps) starting from HP of −89 mV. I-V curves were fitted to the following equation: I = G_max_* (V-V_rev_)/(1 + exp(-(V-V_0.5_)/*k*)), where G_max_ is the maximum slope conductance, V is the test potential, V_rev_ is the extrapolated reversal potential, V_0.5_ is the voltage of half-maximal activation and *k* is the activation slope factor. For pharmacological analysis, cells were depolarized from holding potentials of −59 or −89 mV with 50 ms square pulses to the voltage of maximal inward current (V_max_) with a frequency of 0.1, 0.2 or 1 Hz, as indicated. To mimic SN dopaminergic neuron-like pacemaking activity, action potential waveforms were recorded in substantia nigra dopamine neurons in midbrain slices and used as command voltages with a frequency of 2.5 Hz as described [14]. Cells were continuously perfused by an air pressure-driven perfusion system (BPS-8 or PR-10 Value Control System, ALA Scientific Instruments) with bath solution in the presence or absence of drugs with a flow rate of 350–500 μl/min. Sclareol was purchased from two different vendors (TargetMol, cat# T3020/515–03–7; and Sigma-Aldrich cat# 357995). The latter source was the same as used in the original publication by Wang et al. [11]. For sclareol and cinnarizine (Sigma-Aldrich, cat# C5270) 10 mM stock solutions were prepared in dimethyl sulfoxide (DMSO, Sigma-Aldrich, cat# D8418) and stored at −20°C. Fresh drug dilutions were prepared daily in DMSO by each experimenter. On each experimental day, all chambers subsequently used for pharmacological experiments were tested for the absence of drug effects or perfusion artifacts by switching between bath solutions only. The slope of Ca^2+^ current decrease during pulsing in drug-free solution (“run-down”) was determined by fitting with linear regression at least 5 subsequent data points prior to drug application and subtracted from drug inhibition data. For each cell one or two increasing concentrations of the tested drug were used before the full block of the Ca^2+^ current with 3 μM isradipine (Sigma Aldrich cat# D8418) for L-type channels or 100 μM CdCl_2_ (Sigma Aldrich cat# I6658) for Cav2.3 channels. Concentration for half-maximal inhibition (IC_50_) was determined by fitting the percentage of remaining steady-state current in the presence of drug to the following equation:

$$Y=\mathrm{Bottom}+\frac{\mathrm{top}-\mathrm{Bottom}}{10^{\mathrm{HillSlope}(-\mathrm{LogI}c_{50}+x)}+1}$$

where Y is the percent current or fluorescence in the presence of drug, X is the log of the drug concentration, HillSlope is the slope factor (unit-less), “Top” is 100% of control current in the absence of drug and “Bottom” is the maximal current inhibition.

### Fluorescence-based calcium assay

Cav1.2 and Cav1.3_S_ stable cell lines were seeded onto black, clear bottom 384 well plates (Corning Life Sciences, cat# 3764) and incubated at 37°C, 5% CO_2_ for 48 h to achieve 100% confluency. After induction with doxycycline (1 µg/mL), cells were washed with sterile-filtered washing buffer containing (in mM): 6 KCl, 2 CaCl_2_ x H_2_O, 1.2 MgCl_2_, 144 NaCl, 11 D-glucose monohydrate, 10 HEPES, 4 µM Fluo-4 AM (Thermo Fisher, cat# F14201), pH 7.4. After 45 min incubation (37°C, 5% CO_2_), the cells were washed twice with Krebs-HEPES buffer and once with assay buffer consisting of HBSS (Thermo Fisher, cat# 14025050) with 5 mM HEPES (Sigma, cat# Ho887), pH 7.4. Drug dilutions were freshly prepared daily by dilution of 10 mM DMSO stocks in assay buffer. DMSO 1% was used as control and isradipine as reference compound. Drugs were tested at five to eight concentrations ranging from 100 nM to 50 µM. Assay buffer and drug dilutions were added in a final volume of 50 µL and incubated for 10 minutes before starting the measurement. The measurement was conducted by an Infinite M1000 Pro multimode reader (Tecan) with the following settings: λ_Excitation_ = 485 nm, λ_Emission_ = 520 nm bandwidth 10 nm, Gain 90. Each well was measured for 60 s with a kinetic cycle of one second and injection of KCl solution (90 mM) after 10 s of baseline measurement. Fluorescence increase after injection was calculated (∆F = F_max_ - F_baseline_) and data were normalized to control (1% DMSO) and 10 µM isradipine. At least 3 independent experiments were performed. In each independent experiment drugs were tested in triplicate at each concentration. IC_50_ values were calculated by fitting data as described above.

### Statistics

Data were analyzed using ClampFit 10.2 and ClampFit 10.7 (Axon Instruments), Microsoft Excel, SigmaPlot 12.2 (Systat Software, Inc), GraphPad Prism 8 or 10 (GraphPad software, Inc), and MatLab 9.12.0 (The MathWorks, Inc) software. All values are presented as means ± SD or with 95% confidence intervals (CI) for the indicated number of experiments (n) unless stated otherwise. After normality tests, datasets were analyzed using appropriate statistical tests as indicated. Overall statistical significance was set at *p* < 0.05.

## Results

**Figure 1.**
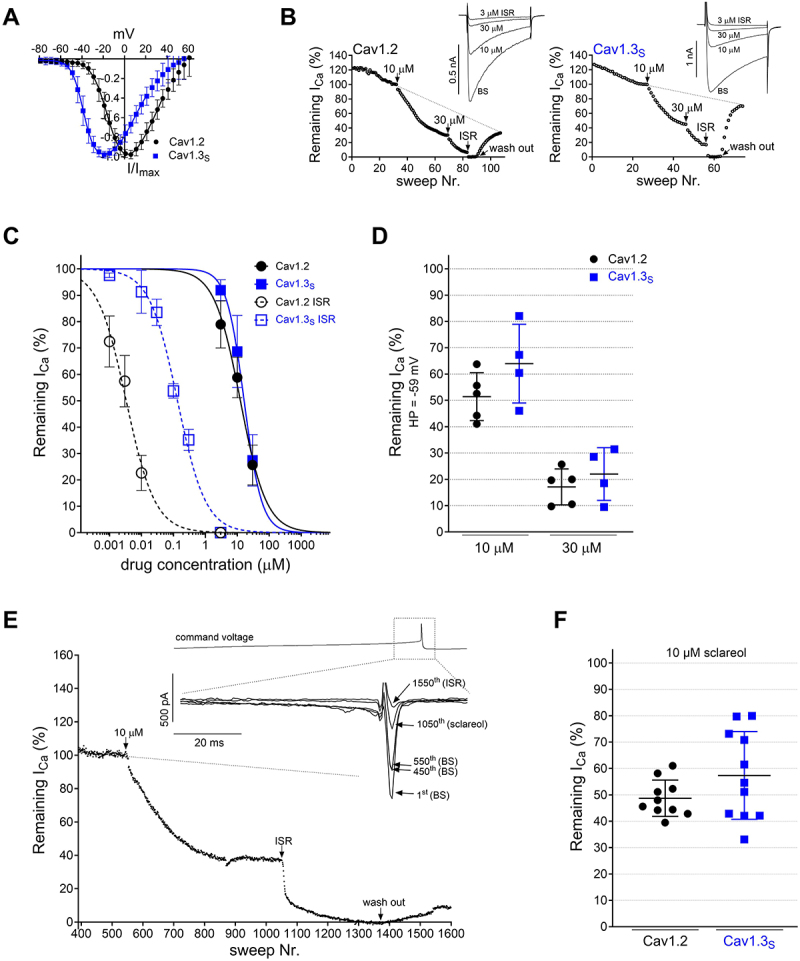
Inhibition of L-type Ca^2+^ channels by sclareol. A. Activation voltage-dependence of stably expressed Cav1.2 (black) and Cav1.3_S_ (blue) channels obtained as described in methods. For statistics see Table 1. B. Time course of Ca^2+^ current inhibition by sclareol. Representative recordings for inhibition of Ca^2+^ currents evoked by 50 ms square pulses from −89 mV to V_max_ at a frequency of 0.1 Hz through Cav1.2 and Cav1.3_S_ upon perfusion with bath solution (BS), sclareol (10 µM and 30 µM) or 3 µM isradipine (ISR) for complete inhibition followed by wash-out with BS. Arrows indicate drug application. The dashed lines indicate the extrapolated Ca^2+^ current decay measured in BS which was used to calculate control current amplitude at each timepoint. C. Concentration-dependent inhibition of Ca^2+^ currents by sclareol (solid lines) and isradipine (dashed lines). IC_50_ values are given in the text. These were obtained by a single fit of all data points (sclareol: Cav1.3 *n* = 20, Cav1.2 *n* = 33; isradipine: Cav1.3_S_ *n* = 21, Cav1.2 *n* = 27). Means are shown ± SD and are from at least *n* = 3 (see Table 2 for statistics), except for Cav1.3_S_ inhibition by 1 nM isradipine (*n* = 2). Data were fitted to the general dose-response equation with top (100%) and bottom (0%) fixed. Statistics (extra sum-of-square-F-test): IC_50_, Cav1.2 vs. Cav1.3_S_: sclareol: *p* > 0.05, isradipine: *p* < 0.001. D. HP of −59 mV: the percentage of remaining current upon perfusion with 10 µM or 30 µM sclareol was not significantly different between Cav1.2 and Cav1.3_S_. Two-way ANOVA. E. Representative experiment illustrating the time course of the inhibition of Cav1.3_S_ currents by 10 µM sclareol during command voltages (inset, grey curve) mimicking substantia nigra dopamine neuron pacemaking (2.5 Hz). As described previously [14], partial inactivation of currents is induced after initiation of this protocol from a HP of −89 mV (1^st^ sweep). As soon as equilibrium under perfusion with BS was achieved (sweeps 450–550, traces shown in inset), sclareol perfusion was started and inhibition quantified at sweep 1050 followed by complete block with 3 µM isradipine. Traces for sweeps 1, 450, 550 and 1050 are shown in the inset. F. Upon perfusion with 10 µM sclareol no statistical difference (two-tailed Student’s unpaired t-test) was observed between Cav1.2 and Cav1.3_S_.

In a recent study, sclareol has been reported as a novel candidate for neuroprotection in PD presumably by preferential inhibition of Cav1.3 L-type channels versus Cav1.2 [11]. This study established a new reporter protein assay in HEK-293 cells, based on a synthetic Ca^2+^-dependent excitation-transcription coupling system for potency assessment. In this assay activation of Cav1 channels by KCl-induced membrane depolarization triggers a cytosolic Ca^2+^ signal, which inhibits or induces reporter protein expression through synthetic gene circuits. Although this assay allowed to quantify drug effects on rodent Cav1.2 and Cav1.3 channel activities, the long exposure to KCl-depolarization and drugs (48 hours) masks the different voltage-dependent gating properties of the channels ([14], see also Table 1, Figure 1(A)) and thus complicate the interpretation of the reported potencies and subtype selectivity of sclareol.

**Table 1.** Voltage-dependent activation parameters of Cav1.2 and Cav1.3_s_ stably expressed in tsA-201 cells in 2 mM Ca^2+^. All data are presented as mean ± SD for the indicated number of experiments (n). V_0.5_, half-maximal activation voltage; *k*, activation slope factor; act thresh, activation threshold.

	V_0.5_ [mV]	*k* [mV]	Act thresh [mV]	
Cav1.2	−11.66 ± 5.22	8.04 ± 1.5	−41.98 ± 4.55	*n* = 30
Cav1.3_S_	−34.94 ± 4.50***	6.65 ± 1.78	−49.82 ± 5.07***	*n* = 34

Statistics: one-way ANOVA with Sidak´s multiple comparison test. ***p < 0.0001.

**Table 2.** Inhibition of Cav1.2, Cav1.3 and Cav2.3 Ca^2+^ channels by sclareol under different experimental conditions. All values are presented as mean ± SD for the number of experiments given in parenthesis. SNDA, substantia nigra dopamine neuron. Transiently expressed hCav1.3_L_ contains alternatively spliced exon 8a.

Construct	Ca^2+^ [mM]	Protocol: HP [mV], Frequency [Hz]	% remaining I_Ca_			
			3 µM	10 µM		30 µM
hCav1.2	2	−59, 0.1	–		51.4 ± 9.1 (5)	17.1 ± 6.8 (5)
hCav1.3_S_	2	−59, 0.1	–		63.9 ± 15.0 (4)	22.0 ± 10.0 (4)
hCav1.2	2	−89, 0.1	79.0 ± 8.9 (6)		58.8 ± 7.7 (8)	25.7 ± 7.6 (6)
hCav1.3_S_	2	−89, 0.1	91.9 ± 4.0 (6)		68.7 ± 13.9 (18)	27.4 ± 9.7 (9)
hCav1.2	2	SNDA-waveform, 2.5	–		48.7 ± 6.9 (10)	–
hCav1.3_S_	2	SNDA-waveform, 2.5	–		36.7 ± 11.2 (8)	–
hCav1.3_L_	2	−89, 0.1	–		66.6 ± 5.2 (8)	–
Cav2.3	2	−89, 0.1	–		36.7 ± 11.2 (8)	–
hCav1.3_L_ transient	15	−89, 0.2	–		75.3 ± 2.9 (4)	–
hCav1.2 transient	15	−89, 0.2	–		70.9 ± 8.8 (4)	–

To directly allow quantification of sclareol selectivity on human Cav1.3_S_ (C-terminal short splice variant) or human Cav1.2 Ca^2+^ channels, we studied its pharmacological properties using whole cell patch-clamp recordings with 2 mM extracellular Ca^2+^ as physiological charge carrier in previously characterized stable cell lines ([14], Table 1, Figure 1(A)). Channels were activated by 50 ms depolarizing pulses from a holding potential (HP) of −89 mV to V_max_ of each cell (Figure 1(A,B)) at 0.1 Hz. After establishing whole-cell contact, perfusion with bath solution resulted in a slow current decrease, which is due to channel inactivation induced by constant pulsing [17]. Sclareol caused a concentration-dependent inhibition of both Cav1.2 and Cav1.3_S_ channels with no evidence for Cav1.3-selectivity (Figure 1(C)) with estimated IC_50_ values of 16.3 µM (95% CI 14.3–19.0) for Cav1.3_S_ and 12.5 µM (95% CI 10.4–15.0) for Cav1.2. These results were obtained with two different sources of sclareol, including one source also used by Wang et al. [11] (see Methods). 3 µM of the DHP isradipine inhibited currents completely (Figure 1(B,C)). For comparison, concentration-response curves were obtained for isradipine as well. Isradipine was more than 100- (Cav1.3) to 1000-fold (Cav1.2) more potent than sclareol under these recording conditions (estimated IC_50_: Cav1.3_S_: 134 nM, 95% CI 112–161; Cav1.2: 3.32 nM, 95% CI 2.75–4.01).

DHPs, such as isradipine, are known to induce state-dependent block of LTCCs [18–20] by preferentially binding to inactivated channel states. To test the possible state-dependent block of sclareol, cells were perfused with 10 and 30 µM sclareol at a more depolarized HP of −59 mV (50 ms test pulses, 0.1 Hz), a HP which significantly increases the apparent potency of DHPs [17]. Both concentrations inhibited Cav1.2 and Cav1.3_S_ to a similar extent and with no increase in potency (Figure 1(D)) as compared to recordings at a HP of −89 mV (Figure 1(C)).

Patch-clamp recordings allow analysis of channel activity and drug effects under more physiological command voltages, including action potential-like waveforms. Given that sclareol has been reported to affect the shape of action potentials in putative SN dopamine neurons [11], we studied its effects on Cav1.3_S_ and Cav1.2 currents elicited during command voltages reproducing action potential waveforms recorded from spontaneously active substantia nigra dopamine neurons (Figure 1(E), inset [14]). Their, on average, more depolarized membrane potential and higher frequency (2.5 Hz) could also help to unmask potential voltage-dependent and frequency-dependent effects of sclareol, respectively. An example of a representative recording of Cav1.3_s_ current is shown in Figure 1(E). As previously shown [14], cells were held at a negative HP (−89 mV) before the start of the action-potential-like stimulation. This initially caused a progressive reduction of peak inward current (not illustrated) to about 50% of initial current during the first 400 sweeps in the absence of drug due to channel inactivation. When equilibrium under perfusion with bath solution was achieved, addition of 10 µM of sclareol reduced current by about 60% (Figure 1(E,F)). Again, this concentration inhibited Cav1.2 and Cav1.3_S_ without significant difference in potencies (Figure 1(F)). Taken together our experiments provide strong evidence for a lack of Cav1 subtype specificity and the absence of a voltage- and use-dependent action of sclareol.

**Figure 2.**
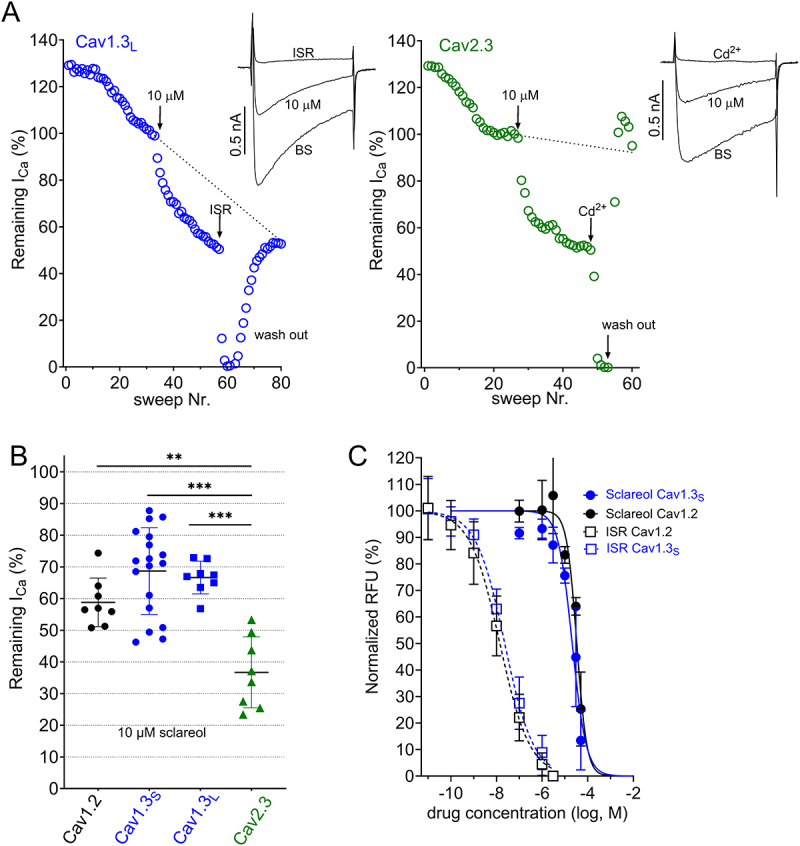
Inhibition of Cav1.3_L_ (blue) and Cav2.3 Ca^2+^ channels (green) by sclareol A. Representative recordings for inhibition with 10 µM sclareol of Ca^2+^ currents through C-terminally long Cav1.3_L_ (left) and Cav2.3 (right) evoked by square pulses from −89 mV to V_max_ at a frequency of 0.1 Hz. Perfusion with bath solution was used to estimate the Ca^2+^ current decay (dashed line). 10 µM sclareol were followed by 3 µM isradipine (ISR) or 100 µM cadmium (Cd^2+^) for complete inhibition and wash-out with bath solution (BS). Arrows indicate when the specified drug was applied. Insets show representative current traces before (BS) drug addition, at steady-state inhibition and after full block by ISR or Cd^2+^. B. 10 µM sclareol inhibited both L-type and Cav2.3 Ca^2+^ channels with slight selectivity for Cav2.3. Statistics: one-way ANOVA with Sidak multiple comparison correction. Means ± SD are shown. C. A fluorescence-based Ca^2+^-flux assay was used to quantify sclareol and isradipine potency on Cav1.2 and Cav1.3_S_ channels depolarized with 90 mM KCl. Sclareol IC_50_ (µM, means, 95% CI): Cav1.2: 33.2 (26.9–39.6, *n* = 22 data points per curve), Cav1.3_S_: 20.9 (15.5–27.3, 18 data points per curve); isradipine IC_50_ (nM, means, 95% CI): Cav1.2 = 14.1 (10.5–18.9, 53 data points per curve), Cav1.3_S_ = 23.0 (18.1–29.3, 52 data points per curve). Each data point is the mean of at least three independent experiments. RFU, relative fluorescent units.

We and others have previously shown that DHP Ca^2+^ channel blockers inhibit Cav1.3_S_ with slightly less potency than the C-terminal long splice variant, Cav1.3_L_ [14,21]. Since both splice variants differ with respect to their biophysical properties [14,15,21] and are both expressed in SN DA neurons [14], we also tested the possibility that sclareol could be a selective inhibitor of Cav1.3_L_. However, 10 µM sclareol inhibited both splice variants with indistinguishable potency (Figure 2(A,B); Table 2).

Importantly, Cav1.3_S_ and Cav1.3_L_ stably expressed in our cell lines contain the alternative exon 8b [22], while Wang et al. used the exon 8a splice variant. In a subset of experiments, we could rule out sclareol´s selectivity toward Cav1.3 containing exon 8a using transiently expressed Cav1.3, which contains exon 8a. Under these experimental conditions (15 mM Ca^2+^ as charge carrier), 10 µM sclareol blocked 25–30% of the Ca^2+^ current of transiently expressed Cav1.3_L_ and Cav1.2 (*p* > 0.05, two-tail Student’s t-test, Table 2).

Since we have recently found that Cav2.3 channels also contribute to the pathophysiology of PD in a mouse PD model [12], we also tested if sclareol inhibits Cav2.3 (R-type) Ca^2+^ channels. Interestingly, Cav2.3 Ca^2+^ currents were even inhibited with significantly higher potency compared to Cav1.2 and to both Cav1.3 splice variants (Figure 2(A,B)).

As the pharmacological assessment of sclareol by Wang et al. [11] was done in a fluorescence-based assay in HEK293-cells and during KCl-depolarization of Cav1.2 and Cav1.3, we repeated our experiments under similar conditions with a fluorescence-based Ca^2+^ flux assay (Figure 2(C)). Again, no evidence for selectivity and high potency was observed (estimated IC_50_: Cav1.3_S_: 20.9 µM (95% CI 15.5–27.3, *n* = 3); Cav1.2: 33.2 µM (95% CI 27.0–39.6, *n* = 4).

**Figure 3.**
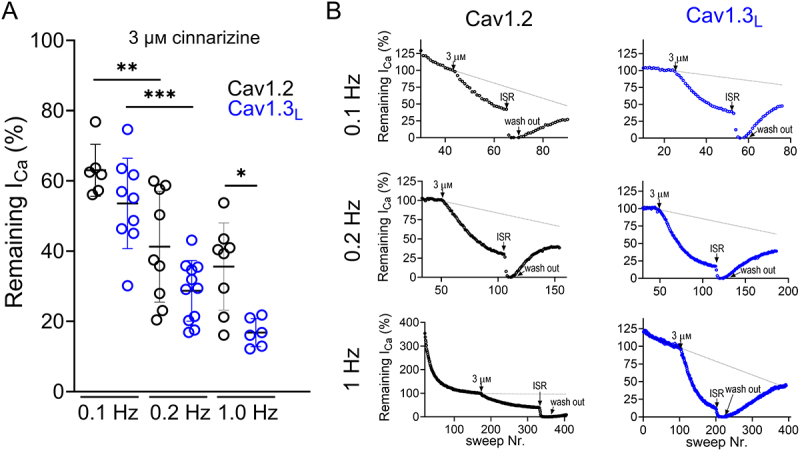
Cav1.2 and Cav1.3_L_ current inhibition by cinnarizine. A. percentage of remaining Ca^2+^ current in Cav1.2 (black) and Cav1.3_L_ (blue) upon perfusion with 3 μM cinnarizine using 50 ms square pulses to V_max_ at 0.1 Hz, 0.2 Hz and 1 Hz. Percentage of remaining current (% mean ± SD): 0.1 Hz, Cav1.2: 63.0 ± 7.4, *n* = 6, Cav1.3_L_: 53.6 ± 12.8, *n* = 9; 0.2 Hz, Cav1.2: 41.3 ± 15.8, *n* = 9, Cav1.3_L_: 28.7 ± 8.6, *n* = 11; 1 Hz, Cav1.2: 35.6 ± 12.4, *n* = 8, Cav1.3_L_: 16.8 ± 3.9, *n* = 6. Statistics: one-way ANOVA with Sidak multiple comparisons test (*, *p* = 0.025; **, *p* = 0.005; ***, *p* < 0.0001). B. Representative traces of Cav1.2 (black) and Cav1.3_L_ (blue) recordings at 0.1 Hz (top), 0.2 Hz (middle) and 1 Hz (bottom) depolarization frequency.

We also investigated the potential Cav1.3-selectivity of the diphenylethylpiperazine derivative cinnarizine. These experiments were motivated by the fact that this drug is clinically used to treat vertigo and motion sickness [23], most likely by inhibiting Cav1.3 channels in vestibular hair cells [24]. Like in auditory inner hair cells, adult vestibular hair cells do not fire action potentials but rather convert head motion-induced movement of their hair bundles into a graded receptor potential (fluctuating between about −70 and −40 mV). This drives glutamate release at their ribbon synapses which triggers action potential activity in their afferent fibers [25]. The Cryo-EM structure of cinnarizine bound to the pore-forming Cav1.3 subunit has recently been solved [10] revealing its binding within the channel pore. Since no systematic comparison of the pharmacological properties of cinnarizine on Cav1.2 and Cav1.3 channels is available, we determined if cinnarizine could act as a subtype-selective inhibitor. Using 0.1 Hz stimulation with 50 ms test pulses and 15 mM Ca^2+^ as charge carrier, 3 µM cinnarizine inhibited Cav1.3_L_ and Cav1.2 currents to a similar extent (46% and 37%, respectively; Figure 3(A)). Given its potential for frequency-dependent channel inhibition due to its binding in the central cavity of the pore domain, we tested if its apparent potency is affected by stimulation frequency. An increase from 0.1 Hz to 0.2 Hz significantly (Figure 3(A,B)) increased apparent potency with 3 µM cinnarizine inhibiting 71% of Cav1.3_L_ and 59% of Cav1.2 currents (Figure 3(A)). Only at 1 Hz stimulation frequency a significant but pharmacologically irrelevant subtype selectivity was observed for Cav1.3_L_.

## Discussion

Using patch-clamp studies with different pulse protocols, we show that sclareol and cinnarizine, two compounds recently claimed to exert their pharmacological effects by the preferential inhibition of Cav1.3 channels, both block Cav1.2 and Cav1.3 with equal potency.

In an attempt to identify novel scaffolds with Cav1.3 L-type Ca^2+^ channel selectivity, Wang et al. recently established a novel cell-based reporter protein-based assay to identify new lead compounds and to be able to screen large compounds libraries [11]. In combination with a virtual screening approach, they identified sclareol in plant essential oils as a potential candidate. While their elegant assay allows the semi-automated testing of a large number of compounds, it does not allow precise estimates of drug potency under defined electrical conditions, which is possible using manual or automated patch-clamping. Our approach allowed the direct quantification of drug effects on channel currents, not confounded by intracellular signaling cascades and subsequent reporter protein expression triggered (or inhibited) by Ca^2+^ influx through L-type channels [11]. Moreover, the protocol used by Wang et al. does not monitor actual membrane potentials, a relevant factor considering the much more hyperpolarized activation threshold of Cav1.3 [1,18] (see Figure 1(A)). Their assay required non-physiological KCl-induced depolarizations in the absence or presence of drug over 48 hours. The reported about 2-fold selectivity of sclareol for Cav1.3 over Cav1.2 (IC_50_ 8.8 vs. 17.7 µM) is of interest, especially considering that currently available Ca^2+^ channel blockers are nonselective or even slightly selective for Cav1.2 (e.g. isradipine, Figure 1(C) [14,26,27]), but not considered pharmacologically relevant [28].

In our patch-clamp recordings we found no evidence for the selective inhibition of Cav1.3-channel currents using standard pulsing protocols from different HP and employing low or higher stimulation frequencies. We also found no evidence for selective inhibition when using a stimulation protocol reflecting the spontaneous action potentials of substantia nigra dopamine neurons.

Wang et al. also provided evidence in their translation-based (CaB-A) reporter assay that sclareol does not inhibit Cav2.2 Ca^2+^ channels, suggesting selectivity for Cav1 L-type versus Cav2 non-L-type channels. In a mouse PD model, Benkert et al. [12] have recently shown that Cav2.3 (R-type) Ca^2+^ channels participate in the generation of somatic pathogenic Ca^2+^ signals in dopamine neurons. This leads to efforts to discover Cav2.3-selective inhibitors for neuroprotection in PD [29]. We show that 10 µM sclareol also inhibited Cav2.3 Ca^2+^ channels with even slightly higher potency than Cav1.2 and Cav1.3. Therefore, our data provide alternative interpretations for the neuroprotective effect of sclareol observed in the 6-OHDA PD-model [11]. While our data do not rule out a contribution of sclareol for neuroprotection by inhibiting Cav1.3, this effect could also involve inhibition of Cav1.2 and/or Cav2.3. Moreover, sclareol neuroprotection may also involve modulation of other ion channels (which has not yet been tested) or even other molecular mechanisms that account for the wide spectrum of bioactivities previously reported for this drug (e.g. anti-tumor, anti-diabetes, and anti-inflammatory effects; for review see Zhou et al. [30]). In any case, our data rule out the possibility of a selective action of sclareol on Cav1.3.

This also holds true for cinnarizine. While its proposed (but never thoroughly characterized) actions on vestibular symptoms and vertigo may be due to the inhibition of Cav1.3 channels in vestibular hair cells, cinnarizine cannot be considered Cav1.3-selective. Its low potency, especially at low electrical activity, and its lack of selectivity may explain its failure to reduce aldosterone production, estimated as aldosterone-to-renin ratio, in a clinical study involving patients with primary aldosteronism [8]. The rationale for this trial was well justified, based on the expected therapeutic benefit of inhibiting Cav1.3 channel-mediated aldosterone secretion [8,31]. Our data suggest that cinnarizine inhibits Cav1.3 channels with relatively low potency and with only limited, and pharmacologically likely irrelevant, selectivity over Cav1.2 only at higher firing frequencies resulting from its frequency-dependent block.

In conclusion, the findings presented in this study offer insights into the pharmacological effects of two compounds proposed to selectively inhibit Cav1.3 channels. Our study revealed no evidence for subtype selectivity among L-type Ca^2+^ channels under different stimulation protocols. Additionally, we provide evidence for Cav2.3 inhibition by sclareol, further demonstrating its lack of selectivity for L-type Ca^2+^ channels.

## Data Availability

The data that support the findings of this study are available from the corresponding author (JS) upon reasonable request.
